# The effectiveness of *Bacopamonnieri* (Linn.) *Wettst*. as a nootropic, neuroprotective, or antidepressant supplement: analysis of the available clinical data

**DOI:** 10.1038/s41598-020-80045-2

**Published:** 2021-01-12

**Authors:** James M. Brimson, Sirikalaya Brimson, Mani Iyer Prasanth, Premrutai Thitilertdecha, Dicson Sheeja Malar, Tewin Tencomnao

**Affiliations:** 1 Age-Related Inflammation and Degeneration Research Unit, Faculty of Allied Health Sciences, Chulalongkorn University, Bangkok, 10330 Thailand; 2 Department of Clinical Chemistry, Faculty of Allied Health Sciences, Chulalongkorn University, Bangkok, 10330 Thailand; 3 Department of Clinical Microscopy, Faculty of Allied Health Sciences, Chulalongkorn University, Bangkok, 10330 Thailand; 4 Siriraj Research Group in Immunobiology and Therapeutic Sciences, Faculty of Medicine Siriraj Hospital, Mahidol University, Bangkok, Thailand

**Keywords:** Clinical pharmacology, Pharmacology, Paediatric research, Dementia, Neurodegeneration, Neurodegenerative diseases, Drug discovery, Neuroscience, Diseases, Health care, Medical research, Molecular medicine

## Abstract

*Bacopa* *monnieri* (Linn.) *Wettst*. has been used in traditional medicine as a drug to enhance and improve memory. In this regard, this study aims to provide *B*. *monnieri*'s efficacy as a neuroprotective drug and as a nootropic against various neurological diseases. Literatures were collected, following Prisma guidelines, from databases, including Scopus, PubMed, Google Scholar, and Science Direct and were scrutinized using a quality scoring system. Means, standard deviations and ‘n’ numbers were extracted from the metrics and analyzed. Jamovi computer software for Mac was used to carry out the meta-analysis. The selected studies suggested that the plant extracts were able to show some improvements in healthy subjects which were determined in Auditory Verbal Learning Task, digit span-reverse test, inspection time task and working memory, even though it was not significant, as no two studies found statistically significant changes in the same two tests. *B*. *monnieri* was able to express modest improvements in subjects with memory loss, wherein only a few of the neuropsychological tests showed statistical significance. *B*. *monnieri* in a cocktail with other plant extracts were able to significantly reduce the effects of Alzheimer’s disease, and depression which cannot be solely credited as the effect of *B*. *monnieri*. Although in one study *B*. *monnieri* was able to potentiate the beneficial effects of citalopram; on the whole, currently, there are only limited studies to establish the memory-enhancing and neuroprotective effects of *B*. *monnieri*. More studies have to be done in the future by comparing the effect with standard drugs, in order to establish these effects clinically in the plant and corroborate the preclinical data.

## Introduction

Neurological disorders include a wide array of problems including neuronal deterioration, cognitive decline, depression, anxiety and have been considered as one of the greatest risks to human health^1^. Even though older adults are more prone to the disease complications, studies reveal that the individual is affected even at a younger age but the pathological outcomes are shown at a later stage^2,3^. Traditional medicinal practices has a long history, and has been practiced worldwide for various diseases including neurological disorders. Due to advancement in scientific research, the role of medicinal plants in treating diseases, their adverse effects, and active compounds exerting the effects have been documented. However, most of the reports for the neuroprotective effects of herbs such as *Bacopa* *monnieri* (Linn.) *Wettst*. are from the pre-clinical studies, which makes it difficult to provide conclusive remarks. In this regard, the current article focusses on the meta-analysis of existing clinical studies on *B*. *monnieri* for its efficacy in treating neurological disorders that helps in connecting statistical significance and draw conclusions.

### *Bacopa monnieri* and its active compounds

**Figure 1:**
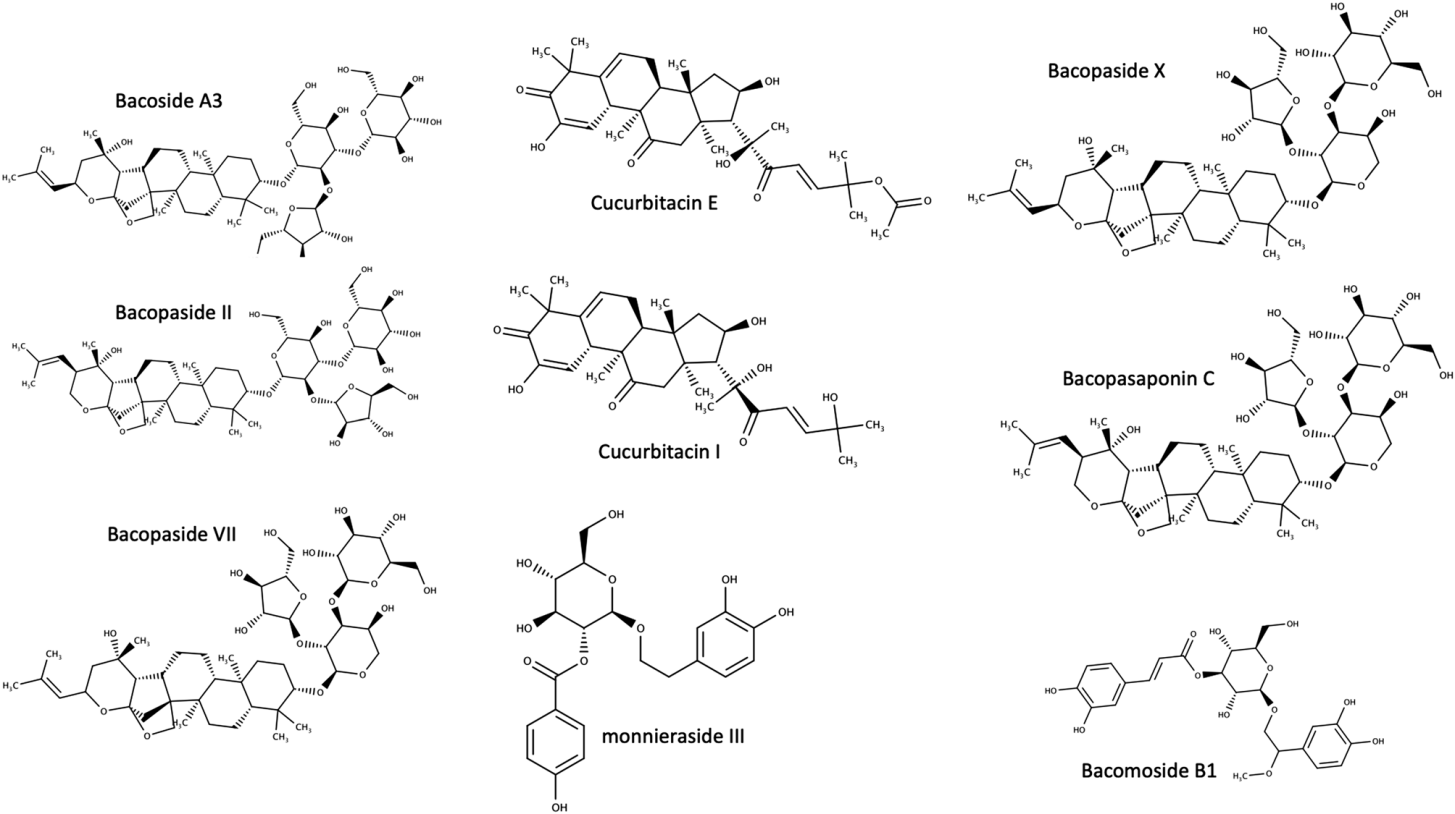
Structures of bacoside compounds that can be isolated from *B*. *monnieri*.

*Bacopa* *monnieri*, a plant belonging to the family Scrophulariaceae, commonly known as Brahmi, occurs naturally throughout south and Southeast Asia. It has a long history in traditional medicine and is known for its memory-enhancing properties as well as reducing anxiety^4^. The herb is currently marketed around the world as a memory enhancer and a blood sugar regulator. The plant has a vast number of active constituent compounds^5^ including several alkaloids and saponins (Fig. 1), with the main active compounds being the steroidal saponins bacosides A (a mixture of bacoside A3, bacopaside II, bacopaside X, and bacopasaponin C)^6^. Though several other medicinal properties of the plant including cardioprotective, hepatoprotective and anticancer activities have been reported, *B*. *monnieri* has been widely used in Ayurveda majorly for its memory boosting property^7–9^.

### Pre-clinical studies of *B*. *monnieri* in Alzheimer’s disease and neuroprotective properties

With the increase in life expectancy throughout the world, possibly due to better healthcare, and access to food and shelter, people are now living longer than any time in history^10^. However, with the increase in the average age of the world’s population, comes new challenges, one of which is neurodegenerative diseases^11^. Alzheimer’s disease is a neurodegenerative disease characterized by β-amyloid plaques, and brain atrophy^12^. The disease is chronic, and symptoms do not usually appear until a significant amount of damage has already occurred in the brain. 60% of dementia cases can be attributed to Alzheimer’s disease. Approximately 44 million people worldwide currently suffer with Alzheimer’s disease, and without the development of a cure or drugs effective at slowing the cognitive decline, this is expected to rise to 135 million by 2050. The cost of caring for patients with Alzheimer’s is equivalent to 1% of the world’s GDP, and then there is the emotional cost on those family members and caregivers who look after the patient as their cognitive ability slowly declines^13,14^.

Studies both in vitro and in vivo suggests the neuroprotective, and nootropic (smart drug) properties and the respective mechanism of action of *B*. *monnieri* against various neuronal insults. The hexane extract of *B*.*monnieri* prevented glutamate induced cell death in HT22 cells by alleviating oxidative and ER stress^15^, while the ethanol extract prevented β-amyloid induced toxicity in cultured neurons by inhibiting acetylcholinesterase enzyme and lipid peroxidation^6^. Studies in transgenic mice bearing human PS-1 and AβPP mutations have shown that both short and long-term treatment of *B*. *monnieri* reduces the amount of β-amyloid found in different regions of the brain^16^. The extract also diminished the neurodegenerative effects of ethylcholine aziridinium ion and colchicine in rats^17,18^, increased cerebral blood flow^19^ and decreased the effects of stress in the brains of rats^20^. Many studies also indicate the antioxidant potential of *B*. *monnieri* that helps in mitigating the adverse effects of oxidative stress in neurons^6,17,18,21–25^. *B*. *monnieri* treatment of rats, under chronic unpredictable stress conditions, stimulates brain-derived neurotrophic factor (BDNF) and the receptor TrkB signaling pathway to activate cAMP response element-binding protein (CREB) resulting in neurogenesis and neuroprotection^26^. Further, the extract also inhibits the release of pro-inflammatory cytokines, namely Tumor necrosis factor-α (TNF-α) and Interleukin-6 (IL-6) from microglial cells and rat brains, thereby protecting from neuroinflammation, a pathological process occurring during neurodegeneration^27,28^. In addition, *B*. *monnieri* extract treatment also reversed aluminium chloride-induced anxiety and scopolamine induced learning and memory deficits in experimental animals^29,30^.

Even so, the neuroprotective and nootropic properties of *B*. *monnieri* and its efficacy in the clinical treatment of various diseases involving neurodegeneration as part of their pathology have not been fully evaluated. Most previous meta-analysis studies have combined the effects on healthy people's memory with that of people with memory complaints and other disorders without concern for specific diseases^10^. Therefore, conclusive evidence for the clinical uses of *B*. *monnieri* as a neuroprotective, antidepressant or nootropic drug in different diseases is warranted for better understanding and more straightforward implementation in further studies.

### Pre-clinical studies regarding depression

Major depression is a neurological condition that is characterized by a minimum of 2 weeks of low mood^31^. Anhedonia, the loss of interest in pleasurable activates, is often a symptom of major depression along with ﻿low self-esteem, lack of energy, and pain without any clear cause^31^. The main hypothesis for the cause of depression, is a lack of monoamines at the synapse in the brain. This is supported by the fact that nearly all antidepressant drugs work to increase the amount of monoamines available at the synapse either by blocking their reuptake, or preventing their break down^32^. At present the commonly prescribed anti-depressant drugs are only effective in 50% of patients, with partial effects seen in 80% of patients^32^. Thus, there is a need for new and improved anti-depressant drugs.

It is important to remember that there is no preclinical model that directly measures a drug’s effect on depression. There are in vivo behavioral animal models that have proved useful in predicting the anti-depressant effects of drugs; however, there is debate about true effectiveness of these models, and whether they are representative of antidepressant effects. One major problem with many of these models is that they are carried out in normal healthy animals, as there is no depressive phenotype model animal. There are protocols that induce depressive line behavior in some of the behavioral tests such as drug withdrawal^33,34^, and stress mechanisms such as chronic unpredictable stress (CUS)^35^. However, these models have been subject to criticism in terms of their reliability and repeatability^36–39^. The Porsolt forced swimming test or behavior despair test^40^ is one of the most common behavioral tests used wherein, animals are subjected to two trials in a beaker of water from which they cannot escape, the first of which lasts 15 min, and the second 24 h later, and lasts five minutes. The time spent not moving except for small movements to stay above the water is measured in the second trial. There are also variations with just one trail^41^. Drugs that have been shown to improve symptoms of major depression, reduce the time spent immobile in this test has traditionally been correlated with improved mood. However, there is some debate about whether increased swimming is a learned behavior or an adaptation to its environment as it becomes more comfortable with its surroundings and less fearful^42^. Indeed, studies have shown that animals placed in a container of water from which they can escape, and thus experience no despair, also show less swimming in the second trial^43^. There are also other contradictions with these despair behavioral models, such as that drugs are administered acutely in these tests, whereas in clinical studies many days or weeks of treatment are required before any antidepressant effects are observed. The reason for this phenomenon is as yet unknown^44^. One possible explanation is the involvement of other receptors and pathways such as the sigma-1 receptor^45,46^, BDNF^47–49^, and inflammation related pathways^50,51^, which over chronic treatment periods increases the plasticity of the neurons and encourages neuron growth^52–55^.

*Bacopa* *monnieri* has been shown to have effects that mimic commonly prescribed antidepressants in the forced swim test (as well as other paradigms such as the tail suspension test and shuttle box testing) in mice and rats^26,56–59^. It is interesting that in one of these studies, using mice suffering from morphine withdrawal as a model of depression, the effect is only seen with chronic treatment^57^. Furthermore, using the CUS to model depression in rats, BDNF expression in the hippocampus of the rats was reduced, which is prevented by *B*. *monnieri* (80 mg/kg). Also, neurogenesis in the Rat hippocampus was improved (compared to the CUS) as measured by 5-bromo-2-deoxyuridine/neuronal nuclei, along with reduction in corticosterone levels^26^. In this study the effects of *B*. *monieri* mimic those of the positive control (imipramine 20 mg/kg) which acts primarily as a sigma-1 ligand^46,60^. Other studies have also shown that CUS induced reductions in BDNF, AKT and CREB expression are reversed by *B*. *monnieri* treatment^61,62^ indicating sigma-1 receptor activation^63–69^ which is hypothesized to be involved in the activities of antidepressant drugs^45,46^. However, there has not been a study directly investigating the role of the sigma-1 receptor in the antidepressant activities of *B*. *monnieri*, although it does seem to be a possible target.

### Aims of this manuscript

The field of herbal medicines and supplements is not as tightly regulated as the pharmaceutical industry. There are many claims made about *B*. *monnieri* such as its memory improving properties mostly based on the available pre-clinical data. This article aims to provide a detailed and up to date review of the neuroprotective and nootropic properties of *B*. *monnieri*, and its efficacy in the treatment of various diseases that have neurodegeneration as part of their pathology. Previous meta-analysis studies have reinvestigated all the diseases jointly and combined the effects on healthy people's memory with that of people with memory complaints and other disorders. These studies could not give a clear picture of the efficacy of *B*. *monnieri*. We have, therefore, tried to separate the different diseases including the memory complaint groups, to provide more conclusive evidence of *B*. *monnieri'*s efficacy as a neuroprotective and nootropic drug.

## Methods

### Literature selection

**Figure 2:**
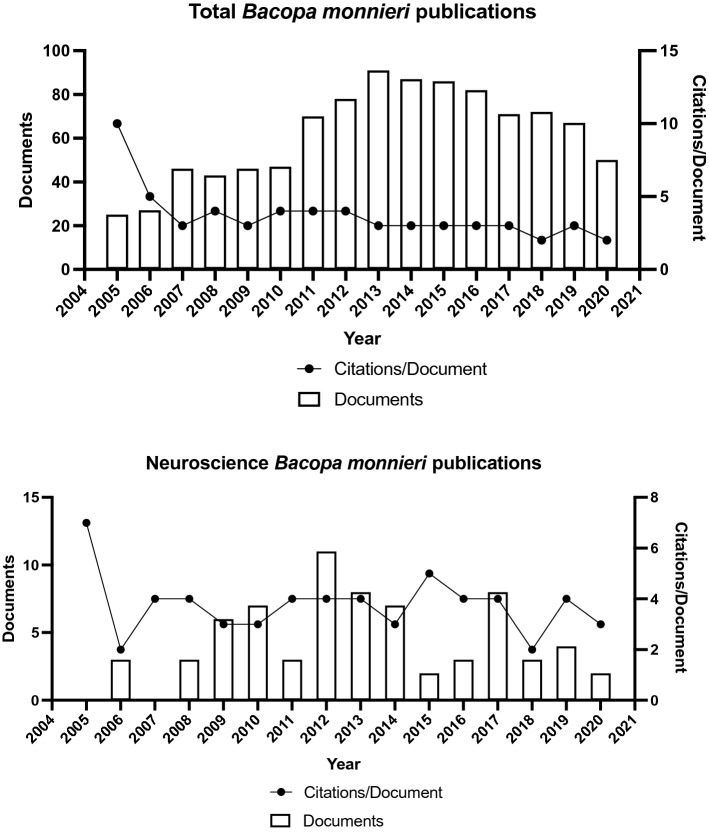
*B*. *monnieri* publication and citation history for the last 15 years (data extracted from Scopus).

**Figure 3:**
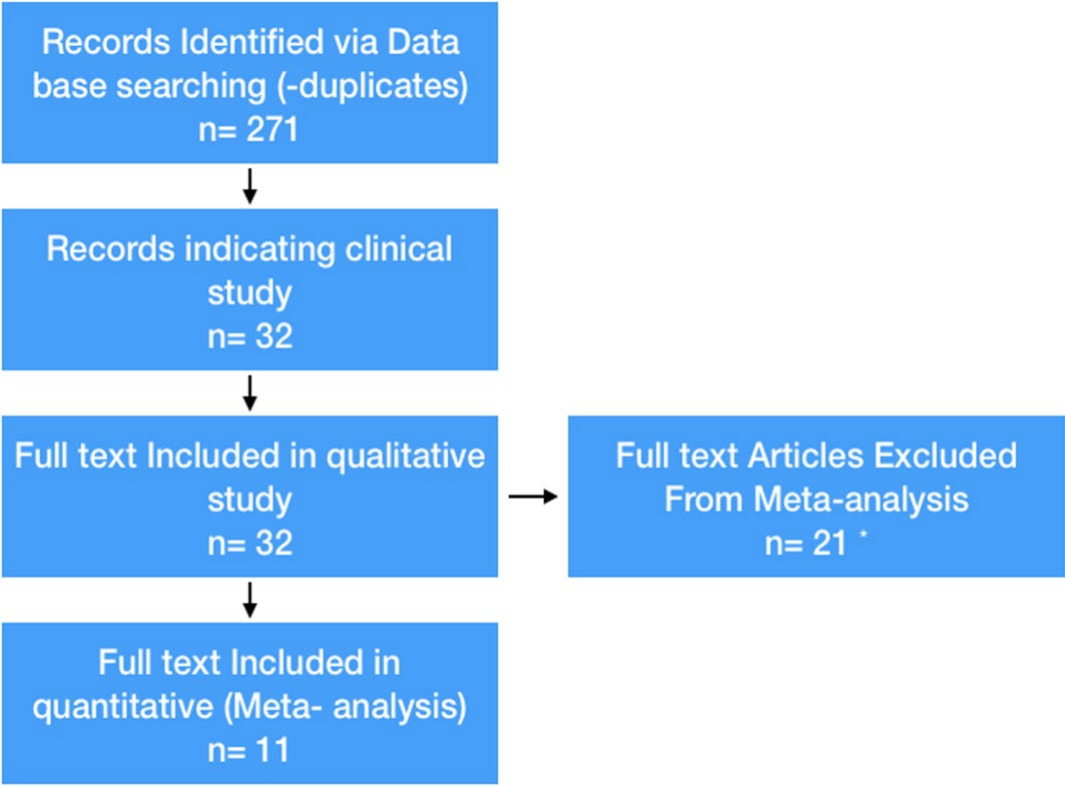
Flow diagram of included studies for meta-analysis of memory improvements caused by *B*. *monnieri* in healthy individuals. *Texts excluded for 1. Multiple herbs included in study 2. Acute study lasting less than six weeks 3. Inappropriate dose used 4. No randomization 5. No control groups 6. Study investigated pharmacological activities other than memory.

Over the past 15 years there has been a lot of interest in *B*. *monnieri* and its potential use in disease treatment, with a total of 838 original articles, 168 review articles and 26 book chapters, of which 74 *B*. *monnieri* publications were related to neuroscience (Fig. 2). A literature search was carried out following Prisma guidelines, searching within Scopus, PubMed, Google Scholar, and Science Direct databases, with no specific timeline, using the search terms, BacoMind, Bacognize, KeenMind, *Bacopa* *monnieri*, *Bacopa* *monniera*, Brahmi, water hyssop, and thyme-leaved Graciela. From the articles found in these searches, the clinical trials were selected for further evaluation. Non-double-blind placebo-controlled studies and studies using *B*. *monnieri* in a formulation combined with other herbs, such as *Ginko* *biloba*, or at a dose less than 200 mg/day were excluded from the meta-analysis (Fig. 3).

### Literature quality scoring

A literature scoring system was employed to rate the quality of each study^70^. One point was awarded for a positive response to each of the following questions; (1) was the study randomized? (2) Was the randomization appropriate? (3) Was the study double-blinded? (4) Was there a description of any withdrawals from the study? (5) Was there a clear description of the inclusion/exclusion criteria for the study? (6) Was there an appropriate control group? (7) Was the dose used appropriate? (8) Were adverse effects monitored and described? (9) Was the method of statistical analysis described? (10) Was there appropriate follow up of patients? (11) Was there a positive control? The total score for each trial falls between 0 and 11, with higher scores being deemed as of higher quality. The literature assessed in this study is summarized in Supplementary Figure S1.

### Statistical analysis

Means and standard deviations were extracted and from the eligible publications and meta-analysis was carried out using Jamovi computer software for Mac^71^. The difference between *B*. *monnieri* treatment and placebo was analyzed using the random effects model, with Dersimonian-liard as the model estimator. Estimated mean difference is presented along with 95% CI, and P value. Heterogeniality statistics are presented with the I^2^ value and its corresponding P value. Data is presented in the form of a Forrest plot showing the weighted means from each study, and the estimated mean difference for the combined studies. Publication bias was assessed using the Rosenthal approach to the Fail-safe N (file draw analysis) and presented with the funnel plot.

## Results

### Quality of included literature and qualitative analysis

Eleven studies^4,72–81^ were selected for inclusion in the meta-analysis, and all scored highly on the study quality scoring system. Each of the studies used a clinically relevant dose, on varying cohort sizes, and measured a range of different neuropsychological tests, before and after the treatment. All 11 were double blinded, randomized and placebo controlled. Two studies had no withdrawals^72,79^, the remaining nine studies all had participants drop out at varying times and for multiple reasons. The dropout rate for each study appeared to be random, between the placebo and the *B*. *monnieri* treated groups, with the exception of one study^77^. Morgan et al.^77^ had a higher rate of dropouts in the *B*. *monnieri* treated group (13 from the *B*. *monnieri* group and 4 from the placebo group). However, the groups were still significantly large enough (*B*. *monnieri* 36—Placebo 45) for the dropouts to have a limited effect on the study. Furthermore, many of the dropouts occurred before the initial treatments, and those that were after the initial baseline test nine were due to side effects and four were for reasons unrelated to the study.

None of the 11 studies carried out the exact same combination of tests, however, all but one^80^ found at least one of their tests to have a statistically significant improvement in at least one of the neuropsychological test used, and thus concluded that their study had shown significant evidence that *B*. *monnieri* improved the cognitive ability of the test subjects, and only one found all their tests had significant improvements compared to control^78^. The neuropsychological tests that were found to have a statistically significant improvement in the *B*. *monnieri* treated patients compared to the placebo are shown in Table 1. Neuropsychological tests that showed no significance in any of the studies were not investigated further, whereas, the studies that were carried out in three or more trials, and where at least one study found significance were investigated. This meant that the following neuropsychological tests were not included in the meta-analysis since less than 3 studies used them; word recognition, picture recognition, spatial working memory, numeric working memory, digit vigilance, inspection time, Trail A, Trail B, and the Stroop task.

**Table 1:** Neuropsychological tests that were found to have a statistically significant improvement in the *B*. *monnieri* treated patients compared to the placebo.

Neuropsychological test	References										
	^4^	^72^	^73^	^74^	^75^	^76^	^77^	^78^	^79^	^80^	^81^
AVLT delayed recall				X		X	X			X	
AVLT learning rate		X				X	X			X	
AVLT forgetting rate		X					X			X	
AVLT proactive interference		X					X			X	
AVLT retroactive interference		X					X			X	
Digit span forwards	X	X	X			X					X
Digit span reverse	X	X	X			X					X
Paired associate learning			X			X					X
Simple reaction time		X			X				X		X
Choice reaction time		X			X				X		X
Logical memory			X					X			X

### Adverse effects of *B*. *monnieri* in the clinical studies included in the meta-analysis

The majority of the studies showed only minor incidence of side effects, with only two studies providing no details of any side effects^76,79^, while the rest included a few incidences of diarrhea, increased stool frequency, nausea, and cramps in the abdomen. These side effects were predominantly seen in the *B*. *monnieri* groups as opposed to the placebo.

## Quantitative analysis

### The effect of *B*. *monnieri* on memory and attention in healthy patients

Across the 11 studies included for the meta-analysis, 645 individuals completed the studies, 323 taking *B*. *monnieri*, and 345 taking the placebo. These were analyzed for the effect of *B*. *monnieri* on memory and attention using meta-analysis (Table 2). There appeared to be very little effect on memory and attention, with the majority of the 11 outcomes analyzed returning no difference between the placebo groups and *B*. *monnieri* groups. The only statistically significant outcomes, wherein, there was a difference between the mean outcomes for the *B*. *monnieri* group and placebo-group was logical memory (Standardized Mean difference 1.11, 95% CI 0.43, 1.80), which suffered from a risk of publication bias (Fail-Safe N = 49). The auditory verbal learning test (AVLT) proactive interference appears to have shown a difference between the *B*. *monnieri* group and placebo, (standardized mean difference 0.62, 95% CI − 0.05, 1.29); however, the difference was not statistically significant (P = 0.071). Furthermore, there was the apparent risk of publication bias [Fail safe N value of 15 (P < 0.001)]. Forest plots and funnel plots along with the full data analysis can be found in Supplementary Figure S2.

**Table 2:** Meta-analysis for the effect of *B*. *monnieri* on memory and attention in healthy people.

Outcomes	*n* Studies	*n* Individuals	Outcome differences		Heterogeneity		Publication bias-fail safe N	Reference
			Weighted mean (95% CI)	P value	I^2^ (%)	P value	Rosenthal (*P* *value*)	
AVLT delayed recall	4	239	0.85 (− 0.22, 1.91)	0.120	93.08	< 0.01	29 (< 0.001)	^74,76,77,80^
AVLT learning rate	4	237	0.50 (− 0.17, 1.16)	0.142	83.53	< 0.01	13 (< 0.001)	^72,76,77,80^
AVLT forgetting rate	3	193	− 0.03 (− 0.31, 0.27)	0.851	0	0.453	0 (0.371)	^72,77,80^
AVLT proactive interference	3	193	0.62 (− 0.05, 1.29)	0.071	80.41	0.006	15 (< 0.001)	^72,77,80^
AVLT retroactive interference	3	193	− 1.3 (− 0.57, 0.31)	0.565	57.17	0.097	0 (0.180)	^72,77,80^
Digit span forwards	5	245	0.07 (− 0.80, 0.91)	0.873	90.28	< 0.01	0 (0.335)	^4,73,76,81^
Digit span reverse	5	245	− 0.04	0.095	78.19	0.001	0 (0.393)	^4,72,73,76,81^
Paired associate learning	3	123	0.31 (− 0.10, 0.72)	0.143	19.9	0.287	1 (0.048)	^73,76,81^
Simple reaction time	4	212	− 0.07 (− 0.36, 0.22)	0.618	0	0.564	0 (0.310)	^72,75,79,81^
Choice reaction time	4	212	− 0.20 (− 0.50, 0.09)	0.179	9	0.518	0 (0.082)	^72,75,79,81^
Logical memory	3	162	1.11 (0.43, 1.80)	0.001	73.23	0.024	43 (0.001)	^73,78,81^

### Qualitative analysis of patients with neurological diseases

Given the interest in the memory-enhancing properties of *B*. *monnieri*, and some promising in vitro and in vivo animal studies^82–85^, it is unsurprising that *B*. *monnieri* would be investigated in patients with memory complaints. Our literature search uncovered four studies that involved clinical trials related to *B*. *monnieri*, two of which; however, included other herbs in their formulation^86,87^. One used a dose of 250 mg/day which is still likely to be clinically relevant^73^. Two studies were not randomized or double-blind and had no valid controls^88,89^ as such, they were, assessed accordingly.

Cicero et al.^87^ showed significant cognitive improvements in patients with mild cognitive impairment when treated with a multi-herb formulation, which included *B*. *monnieri*, l-theanine, *Crocus* *sativus*, copper, folate, and vitamins B and D^87^. When compared to the placebo there were statistically significant improvements in the Mini-Mental State Examination (MMSE), Perceived Stress Questionnaire (PSQ) and Index and Self-Rating Depression Scale (SRDS).

### *B*.* monnieri* and treatment of Alzheimer's disease

Our literature search found only two studies that investigated the effects of *B*. *monnieri* on patients diagnosed with Alzheimer's disease^86,89^. However, the Goswami et al.^89^ study was an open label, non-randomized, non-controlled study, thus scoring very low on the literature scoring analysis and the formulations used in the Sadhu et al.^86^ study included other herbal extracts, and thus, any positive effects seen in this study cannot be attributed directly to *B*. *monnieri*. Therefore, we cannot carry out a meta-analysis for *B*. *monnieri* in Alzheimer’s treatment since there is only one study that fits the inclusion criteria. Despite this, we feel the two studies warrants inclusion and quantitative discussion within this review, since there are so few studies in Alzheimer’s patients with *B*. *monnieri* and Sadhu et al.^86^ is the only study to compare the herbal formulation to that of an already approved Alzheimer's drug (donepezil). Sadhu et al.^86^ investigated a cohort of Alzheimer's patients between the ages of 60 and 75, as well as a group of healthy patients in the same age group. The study was also conducted over 12 months, rather than the 12 weeks like most of the other studies described here. The study found a significant improvement in the neuropsychological exams [MMSE, digital symbol substitution, delayed word recall score, attention span, frequently asked questions (FAQ) score, and depression score] in the healthy patients when comparing between the drug formulation and the placebo. Furthermore, in Alzheimer's patients, the improvement was comparable to that of donepezil in the MMSE, dementia screening scale (DSS), word recall immediate, word recall delayed, attention span, FAQ score, and depression scores^86^.

The study also investigated various biochemical markers for inflammation and oxidative stress. Healthy patients given the drug *vs*. healthy patients given the placebo found significant reductions in pro-inflammatory cytokines, including IL-6 and TNF-α. There were also significant reductions in homocysteine, C-reactive protein (CRP), superoxide dismutase (SOD), and glutathione peroxidase (GPx) activity in the healthy patients given the test formulation compared to the placebo. Furthermore, there were significant improvements in oxidative stress and inflammation markers in the herb treated group, compared to the donepezil treated group, with reductions in homocysteine, CRP, and TNF-⍺, as well as reductions in SOD, GPx, thiobarbituric acid reactive substances (TBARS) and an increase in glutathione (GSH). The study concludes by arguing that the combined herbal drug, with its multiple active compounds, acts on multiple targets such as glutamatergic, gamma-amino butyric acid (GABA)-ergic, dopaminergic, noradrenergic and serotonergic receptors, giving results comparable to that of donepezil (an acetylcholinesterase inhibitor and possible sigma-1 receptor ligand). Furthermore, the multi-herbal drug improves on donepezil by reducing oxidative stress and inflammation in Alzheimer's disease patients^86^.

Goswami et al.^89^ investigated the effect over 6 months of a daily dose of 600 mg *B*. *monnieri* in 39 patients formally diagnosed with Alzheimer’s disease. The study found mild statistically significant increase in performance in various aspects of the MMSE tests, and concludes that *B*. *monnieri* is beneficial in Alzheimer’s disease patients. However, since this study is neither placebo-controlled or randomized and does not compare to a positive control such as donepezil, it is not possible to assess the true value of *B*. *monnieri* in Alzheimer’s disease patients.

### *B*.* monnieri* and effects on depression

Multiple studies in animal models have identified *B*. *monnieri* as a potential antidepressant, having similar effects to fluoxetine in stress models of depression^61,90–92^. Our literature search uncovered four randomized placebo-controlled double-blind studies, using *B*. *monnieri* as a single herb intervention^4,74,77,80^, and two studies^86,87^ using a multi-herb intervention, which included depression or anxiety metrics in their analyses. However, it is essential to note that these studies were carried out on patients who did not necessarily suffer from clinically diagnosed depression. We found only one clinical study that investigated patients with depression; this study was not a placebo-controlled study^93^, rather, it compared patients treated with citalopram (40 mg), to patients treated with *B*. *monnieri* (300 mg twice per day) and citalopram (40 mg) for 4 weeks.

There was no combination of these studies that used the same tests to study depression and anxiety; thus, no meta-analysis can be carried out. Only one of the single herb intervention studies exhibited a statistically significant effect on depression and anxiety compared to the placebo^74^. However, the antidepressant effect was small despite being statistically significant. Sathyanarayanan et al. used the same anxiety measure as Calabrese et al. and found no significant difference between *B*. *monnieri* and placebo-treated groups^74,80^. Both Morgan et al. (2010) and Roodenrys et al. (2002) found no significant change in depression scores using the Hamilton rating scale for depression (HAMD) and depression anxiety stress scales-depression (DASS-D) analyses, respectively^4,77^. The two multi-herb studies found that there were statistically significant changes in stress^87^ and depression^86^. The Sadhu et al.^86^ study showed the most substantial improvement in depression scores, in both healthy patients (*vs*. placebo) and in the patients who have Alzheimer's disease. The Alzheimer's disease patients had a much higher geriatric depression scale (GDS) score than the healthy volunteers, at the start of the study. When treating the patients with *B*. *monnieri* over 12 weeks, the score was reduced to levels similar to that of healthy volunteers, whereas donepezil had no significant effect of GDS.

In the one study that did investigate patients diagnosed with anhedonia (a common symptom of depression as well as other neurological disorders) there was shown significant improvements in the *B*. *monnieri* group compared to citalopram alone in the Hamilton depression rating scale, Snaith–Hamilton Pleasure Scale (SHAPS), and strength and difficulties questionnaire^93^.

## Discussion and conclusions

Each of the clinical studies in healthy people measured an array of different neuropsychological tests; however, no two studies found statistically significant changes across the same neuropsychological tests. Furthermore, the majority of the studies only found one or two measures of memory to have a statistically significant difference, and even then, these changes were small.

There were two clinical studies with statistically significant improvements in memory from older people who complained of memory loss, without any sign of Alzheimer's or dementia. However, the improvements were modest, and only a few of the many neuropsychological tests showed statistically significant changes; furthermore, as with the healthy volunteers, no two studies found significant differences in the same tests. These studies could suffer from familywise (type one) errors, as so many variables were being investigated, there is a greater chance of finding significance in at least one of them.

Only one double blind placebo-controlled study has tested the effects of *B*. *monnieri* in patients with Alzheimer's; however, that study used a poly-herb formulation, and thus, any effects observed cannot be directly attributed to *B*. *monnieri* per se. The poly herb formulation has showed some effects against placebo in healthy patients and appeared to be equal to the standard acetylcholinesterase inhibitor donepezil in Alzheimer's patients, with the exception of the depression scores where *B*. *monnieri* gave a substantial improvement compared to Alzheimer's patients treated with donepezil, returning the depression score close to that of the healthy aged adults. One uncontrolled open label study investigated beneficial effects in Alzheimer’s patients, and found there to be some positive effects. However, since this study was not placebo controlled, it is not possible to rule out the placebo effect. Furthermore, it is not possible to evaluate the extent of the effect of *B*. *monnieri* in Alzheimer’s patients in this study as it does not compare to current standard drugs for Alzheimer’s disease such as donepezil.

The majority of studies that investigated *B*. *monnieri'*s influences on depression and anxiety, did so on patients that had not been clinically diagnosed with depression. It appears that each study that has included depression and or anxiety has done so as an afterthought, thus reducing the validity of the findings. Furthermore, since most of the above studies used *B*. *monnieri* to treat patients that had not been clinically diagnosed with depression or anxiety, we cannot conclusively say that it would not be beneficial. Particularly as the one study that did investigate patients with anhedonia did appear to potentiate the effects of citalopram. Without placebo-controlled studies on patients suffering from depression, which compare to a clinical antidepressant such as fluoxetine, we cannot rule out the anti-depressant properties of *B*. *monnieri*, particularly in the case of Alzheimer’s patients suffering from depression.

In conclusion, there is little to no clinical evidence to suggest that *B*. *monnieri* improves the memory of healthy adults and adults with age related memory complaints. Furthermore, the clinical evidence for the treatment of Alzheimer's and depression with *B*. *monnieri* is sparse; since very few studies have investigated Alzheimer's disease, with a single herb formulation of *B*. *monnieri*, and none have investigated patients with depression. Moreover, larger extensive long-term studies that go head to head with current standard drugs are required to determine whether *B*. *monnieri* is a viable alternative medicine in the treatment of the diseases discussed above. These studies should be standardized and carry out the same set of neuropsychological tests, such that future meta-analysis studies may be carried out.

## Supplementary Information

Supplementary Information 1.

Supplementary Information 2.
