# Supplementary material for: The effectiveness of Bacopamonnieri (Linn.) Wettst. as a nootropic, neuroprotective, or antidepressant supplement: analysis of the available clinical data
Source: Sci Rep. 2021 Jan 12;11:596. doi: 10.1038/s41598-020-80045-2 (PMC7803732; doi:10.1038/s41598-020-80045-2)
Supplement: Supplementary file 1 — Supplementary Information 1. [file 41598_2020_80045_MOESM1_ESM.pdf]

The effectiveness of *Bacopa monnieri* (Linn.) *Wettst.* as a nootropic, neuroprotective, or antidepressant supplement: Analysis of the available clinical data.

James M. Brimson, Sirikalaya Brimson, Mani Iyer Prasanth, Premrutai Thitilertdecha, Dicson Sheeja Malar, Tewin Tencomnao.

Supplementary Table 1. The literature summarized within this analysis before excluding polyherbal formulations and acute studies

**Formulation (Dose used)**	**Candidates per group (*n*)**	**Study type**	**Results and statistically significant outcomes (P<0.01)**	**Reference**
KeenMind (300-450 mg/day)	KeenMind *n*=37<br>Placebo *n*=39	Randomized, double-blind, placebo-controlled; memory in middle-aged otherwise healthy people	No significant outcomes at P<0.01 from 11 tests	1
KeenMind (300 mg/day)	KeenMind *n*=23<br>Placebo *n*=23	Randomized, double-blind, placebo-controlled; memory in young to middle-aged, otherwise heathy people	Reduction in anxiety. No significant outcomes at P<0.01 from 15 other tests	2
KeenMind (320 or 640 mg/day)	KeenMind *n*=20<br>Placebo *n*=20	Randomized, double-blind, placebo-controlled: memory in otherwise healthy people over 55 years old	No significant outcomes at P<0.01 from 7 types of test	3
Dried *B. monnieri* extract (300 mg/day)	Bacopa *n*=24<br>Placebo *n*=24	Randomized, double-blind, placebo-controlled; memory, anxiety and depression in otherwise healthy elderly people	Improvements in delayed recall and Stroop test. Not significant in 3 other tests	4
KeenMind (300 mg/day)	KeenMind *n*=29<br>Placebo *n*=32	Randomized, double-blind, placebo-controlled; memory in young to middle-aged, otherwise heathy people	No significant outcomes at P<0.01 from 19 tests	5
BacoMind (450 mg/day)	BacoMind *n*=23<br>Placebo *n*=21	Randomized, double-blind, placebo-controlled; memory in otherwise healthy elderly people	Improvements in digit-span backwards, & list learning-delayed recall. Not significant in 21 other tests	6
BacoMind (300 mg/day)	BacoMind *n*=49<br>Placebo *n*=49	Randomized, double-blind, placebo-controlled; memory in otherwise healthy elderly people	Improvements in AVLT and AVLT delayed recall.<br>Not significant in 11 other tests	7
Extract *B. monnieri* whole plant (100 mg) powder *B.*	Bacopa *n*=41	Randomized, placebo controlled in healthy	No significant outcomes at P<0.01 from 3 tests	8

monnieri whole plant (650 mg)	Placebo *n*=43	participants between the age of 30 and 42.		
B. monnieri standardized tablet (300 or 600 mg)	Bacopa 300 mg *n*=20<br>Bacopa 600 mg *n*=20<br>Placebo *n*= 20	Randomized, double-blind, placebo-controlled; memory in young to middle-aged, otherwise heathy people	Significant improvements in power of attention, continuity of attention, speed of memory and quality of memory	9
BacoMind (450 mg/day)	BacoMind *n*=33<br>Placebo *n*=33	Randomized, double-blind, placebo-controlled; memory and anxiety in middle-aged otherwise healthy people	No significant outcomes at P<0.01 from 14 tests	10
B. monnieri, H. rhamnoides and *D. bulbifer*a combined (500 mg/day)	(normal patients) Bacopa *n*=56<br>Placebo *n*=41<br>(SADT)<br>Donepezil *n*=43<br>Bacopa *n*=61	Randomized, double-blind, placebo-controlled + standard drug (donepezil): memory and depression in Alzheimer's diseases patients	Improvements in MMSE, DSS, delayed recall, attention span and depression.	11
Polyherbal formulation containing *B. monnieri* dry extract (320 mg), L-theanine (100 mg), *C. sativus* 30 mg, + vitamins and minerals	Bacopa *n*=15<br>Placebo *n*=15	Randomized, double-blind, placebo-controlled. Depression, memory and stress in elderly people with self-reported memory loss.	No significant outcomes at P<0.01 from 3 types of test	12
H. pluvialis (74 mg)<br>Astaxanthin (2 mg)<br>*B. monnieri* dry extract 20% (100 mg)<br>Bacosides (20 mg)<br>Phosphatidylserine (extracted from soy) (30 mg)<br>Vitamin E (30 mg)	Herbal supplement *n*=104	Non-comparative, exploratory clinical study on Alzheimer's patients.<br>Non-placebo-controlled	Improvements in Das-cog, and clock drawing test	13
Bacognize (300 mg/day)	Baconize *n*=39	Alzheimer’s disease patients. Open label, uncontrolled, non-randomized	Improvements in MMSES	14
Citalopram (40 mg) alone or Citalopram +*B. monnieri* (300 mg)	Citalopram *n*=23<br>Citalopram + Bacopa *n*=19	Randomized, comparison of anhedonia in patients with unsatisfactory results after 4 weeks of citalopram treatment	Bacopa improved citalopram’s effects in SHAPS, HAM-D and SDQ. P<0.05	15

Mentat polyherbal formulation, 13 herb extracts including *B. monnieri,* (136 mg) and *C. asiatica* (70 mg), and 11 other powdered herbs	Placebo *n*=20<br>Mentat *n*=20	Double-blind, placebo-controlled study; Behavior of children	No significant outcomes at P<0.01	16
Mentat polyherbal formulation, 13 herb extracts including *B. monnieri,* (136 mg) and *C. asiatica* 70 mg, and 11 other powdered herbs	Placebo *n*=30<br>Mentat *n*=30	Randomized, double-blind, placebo-controlled trial. Behavior in children with ADHD	No significant outcomes at P<0.01	17
B. monnieri (300 mg; 215 mg) and *Ginkgo biloba* (120 mg; 260 mg)	Bacopa/Ginko *n*=44<br>Placebo *n*= 41	Randomized, double-blind, placebo-controlled, cognitive performance in otherwise healthy young to middle-aged people.	No significant outcomes at P<0.01 from 17 tests	18
BacoMind (300-450 mg/day)	BacoMind *n*=23	Phase I safety study	Mild adverse events related to gastrointestinal system	19
Manas Niyamak Yoga. Poly herbal formulation including *B. monnieri*, *C. asiatica*, and others	Placebo *n*=10<br>Manas Niyamak Yoga *n*=10	Randomized, double-blind, placebo-controlled study, measuing ADHD in children	Improvements in division of attention and reaction time	20
Brahmi (250 mg) twice a day after meals	Bacopa *n*=20<br>Caffeine *n*=20	Randomized 40 healthy male medical student volunteers in the age group of 25 ± 5 years.<br>No placebo control	Significant improvements in simple reaction time, multiple choice reaction time, digit cancellation task (DCT), mental arithmetic task (MAT) and memory test (MT)	21
Nurture & Clarity. Poly herbal formulation including *B. monnieri, Paeoniae alba*, and others	Placebo *n*=40<br>Treatment *n*=80	Randomized, double-blind, placebo-controlled trial. Measuring ADHD in children	Improvements in response time and variability	22
Memomet polyherbal formulation: *B. monnieri* (125 mg), *C. pleuricaulis* (100 mg) and *C. asiatica* (100 mg).	Placebo *n*= 30<br>Memomet *n*=56	Randomized, double-blind, placebo-controlled trial. Behavior in children with ADHD	No significant outcomes at P<0.01	23
KeenMind (320 or 640 mg/day)	Not reported	Acute randomized, double-blind, placebo-controlled: mood anxiety and depression in otherwise heathy young to middle-aged	No significant outcomes at P<0.01 from 5 types of test	24

KeenMind (320 or 640 mg/day)	Not reported	Acute randomized, double-blind, placebo-controlled, cognitive performance in otherwise healthy young to middle-aged people.	Improvements in reverse counting in 3s and 7s and improvements in stress. No significant improvements in rapid visual information processing task (RVIP)	[25]
BacoMind (225 mg/day)	BacoMind *n*=24	Open label study: ADHD in children	No significant outcomes at P<0.01	[26]

References

1 Roodenrys, S. *et al.* Chronic Effects of Brahmi (*Bacopa monnieri*) on Human Memory. *Neuropsychopharmacology* **27**, 279-281 (2002).

2 Stough, C. *et al.* The chronic effects of an extract of *Bacopa monniera* (Brahmi) on cognitive function in healthy human subjects. *Psychopharmacology* **156**, 481-484, doi:10.1007/s002130100815 (2001).

3 Raghav, S., Singh, H., Dalal, P. K., Srivastava, J. S. & Asthana, O. P. Randomized controlled trial of standardized *Bacopa monniera* extract in age-associated memory impairment. *Indian Journal of Psychiatry* **48**, 238-238, doi:10.4103/0019-5545.31555 (2006).

4 Calabrese, C. *et al.* Effects of a Standardized *Bacopa monnieri* Extract on Cognitive Performance, Anxiety, and Depression in the Elderly: A Randomized, Double-Blind, Placebo-Controlled Trial. *The Journal of Alternative and Complementary Medicine* **14**, 707-713, doi:10.1089/acm.2008.0018 (2008).

5 Stough, C. *et al.* Examining the Nootropic Effects of a special extract of *Bacopa monniera* on Human Cognitive Functioning: 90 day Double-Blind Placebo-Controlled Randomized Trial. *Phytotherapy research* **24**, 54-59, doi:10.1002/ptr (2008).

6 Barbhaiya, H. C. *et al.* Efficacy and tolerability of BacoMind on memory improvement in elderly participants -A double blind placebo controlled study. *Journal of Pharmacology and Toxicology* **3**, 425-434, doi:10.3923/jpt.2008.425.434 (2008).

7 Morgan, A. & Stevens, J. Does *Bacopa monnieri* Improve Memory Performance in Older Persons? Results of a Randomized, Placebo-Controlled, Double-Blind Trial.

The Journal of Alternative and Complementary Medicine **16**, 753-759, doi:10.1089/acm.2009.0342 (2010).

8 Mandal, A. K., Hedge, S. & Patki, P. S. A clinical study to evaluate the efficacy and safety of Bacopa caplets in memory and learning ability: A double blind placebo controlled study. *Australian Journal of Medical Herbalism* **23**, 122-125 (2011).

9 Peth-Nui, T. *et al.* Effects of 12-Week *Bacopa monnieri* Consumption on Attention, Cognitive Processing, Working Memory, and Functions of Both Cholinergic and Monoaminergic Systems in Healthy Elderly Volunteers. *Evidence-Based Complementary and Alternative Medicine* **2012**, 1-10, doi:10.1155/2012/606424 (2012).

10 Sathyanarayanan, V. *et al.* Brahmi for the better? New findings challenging cognition and anti-anxiety effects of Brahmi (*Bacopa monniera*) in healthy adults. *Psychopharmacology* **227**, 299-306, doi:10.1007/s00213-013-2978-z (2013).

11 Sadhu, A. *et al.* Management of Cognitive Determinants in Senile Dementia of Alzheimer's Type: Therapeutic Potential of a Novel Polyherbal Drug Product. *Clinical Drug Investigation* **34**, 857-869, doi:10.1007/s40261-014-0235-9 (2014).

12 Cicero, A. F. *et al.* Short-Term Impact of a Combined Nutraceutical on Cognitive Function, Perceived Stress and Depression in Young Elderly with Cognitive Impairment: A Pilot, Double-Blind, Randomized Clinical Trial. *The journal of prevention of Alzheimer's disease* **4**, 12-15, doi:10.14283/jpad.2016.10 (2017).

13 Zanotta, D., Puricelli, S. & Bonoldi, G. Cognitive effects of a dietary supplement made from extract of *Bacopa monnieri*, astaxanthin, phosphatidylserine, and vitamin E in subjects with mild cognitive impairment: A noncomparative, exploratory clinical study. *Neuropsychiatric Disease and Treatment* **10**, 225-230, doi:10.2147/NDT.S51092 (2014).

14 Goswami, S. *et al.* Effect of *Bacopa monnieri* on cognitive functions in Alzheimer's disease patients. *International Journal of Collaborative Research on Internal Medicine and Public Health* **3**, 285-293 (2011).

15 Micheli, L. *et al.* *Bacopa monnieri* as augmentation therapy in the treatment of anhedonia, preclinical and clinical evaluation. *Phytotherapy Research* **34**, 2331-2340, doi: 10.1002/ptr.6684 (2020).

16 Patel, R. B. Experience with Mentat in Hyperkinetic Children. *Probe* **3**, 271-274 (1991).

17 Kalra, V. & Kulkarni, K. S. A randomized double blind placebo-controlled drug trial with Mentat in children with Attention Deficit Hyperactivity Disorder. *Neurosciences Today* **4**, 223-227 (2002).

18 Nathan, P. J. *et al.* Effects of a combined extract of Ginkgo biloba and Bacopa monniera on cognitive function in healthy humans. *Human Psychopharmacology* **19**, 91-96, doi:10.1002/hup.544 (2004).

19 Pravina, K. *et al.* Safety evaluation of BacoMind™ in healthy volunteers: a phase I study. *Phytomedicine* **14**, 301-308 (2007).

20 Ojha, K., Kumar, A. & Rai, M. Clinical Study on the Role of an Ayurvedic. *Ayurveda* **1**, 39-47 (2007).

21 Raina, R. S. *et al.* The Psychomotor Effects Of Brahmi And Caffeine On Healthy Male Volunteers RAINA. *Journal Of Clinical and Diagnostic Research* **3**, 1827-1835, doi:10.1016/j.wem.2013.09.006 (2009).

22 Katz, M., Levine, A. A., Kol-Degani, H. & Kav-Venaki, L. A compound herbal preparation (CHP) in the treatment of children with ADHD: A randomized controlled trial. *Journal of Attention Disorders* **14**, 281-291, doi:10.1177/1087054709356388 (2010).

23 Dutta, B. *et al.* A study of evaluation of safety and efficacy of memomet, a multi herbal formulation (memomet) in the treatment of behavioural disorder in children. *International Journal of Research in Pharmaceutical Sciences* **3**, 282-286 (2012).

24 Benson, S. *et al.* Erratum: An acute, double-blind, placebo-controlled cross-over study of 320-mg and 640-mg doses of *Bacopa monnieri* (CDRI 08) on multitasking stress

reactivity and mood (Phytotherapy Research (2014) 28 (551-559) DOI: 10.1002/ptr.5029). *Phytotherapy Research* **29**, 1676-1676, doi:10.1002/ptr.5439 (2015).

25 Downey, L. A. *et al.* An acute, double-blind, placebo-controlled crossover study of 320 mg and 640 mg doses of a special extract of *Bacopa monnieri* (CDRI 08) on sustained cognitive performance. *Phytotherapy Research* **27**, 1407-1413, doi:10.1002/ptr.4864 (2013).

26 Dave, P. U., Dingankar, S. R. & Saxena, V. S. An Open-label Study to Elucidate the Effects of Standardized *Bacopa monnieri* Extract in the Management of Symptoms of Attention-deficit Hyperactivity Disorder in Children. *Advances* **28**, 10-15 (2014).
