# Supplementary material for: The effectiveness of Bacopamonnieri (Linn.) Wettst. as a nootropic, neuroprotective, or antidepressant supplement: analysis of the available clinical data
Source: Sci Rep. 2021 Jan 12;11:596. doi: 10.1038/s41598-020-80045-2 (PMC7803732; doi:10.1038/s41598-020-80045-2)

The effectiveness of *Bacopa monnieri* (Linn.) *Wettst.* as a nootropic, neuroprotective, or antidepressant supplement: Analysis of the available clinical data.

James M. Brimson, Sirikalaya Brimson, Mani Iyer Prasanth, Premrutai Thitilertdecha, Dicson Sheeja Malar, Tewin Tencomnao.

Supplementary Figure 2. The meta-analysis of neuropsychological tests carried out on healthy patients treated with *B. monnieri* or placebo. Forest plots and funnel plots along with the full data analysis

AVLT Delayed Recall

Random-Effects Model (k = 4)

	Estimate	se	Z	p	CI Lower Bound	CI Upper Bound
Intercept	0.845	0.543	1.56	0.120	-0.219	1.910
	.	.	.	.	.	.

Note. Tau² Estimator: DerSimonian-Laird

Heterogeneity Statistics

Tau	Tau²	I²	H²	R²	df	Q	p
1.041	1.0838 (SE= 1.0106)	93.08%	14.444	.	3.000	43.332	< .001

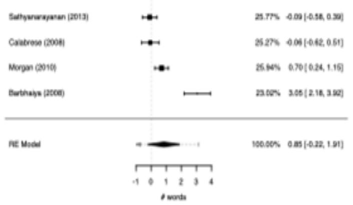

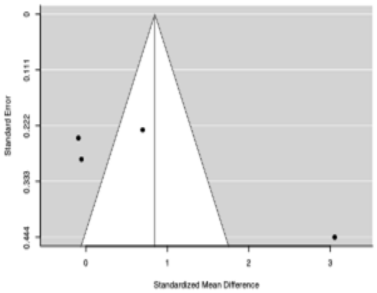

AVLT Learning Rate

Random-Effects Model (k = 4)

	Estimate	se	Z	p	CI Lower Bound	CI Upper Bound
Intercept	0.497	0.338	1.47	0.142	-0.166	1.159
	.	.	.	.	.	.

Note. Tau² Estimator: DerSimonian-Laird

Heterogeneity Statistics

Tau	Tau²	I²	H²	R²	df	Q	p
0.615	0.3778 (SE= 0.379)	83.53%	6.070	.	3.000	18.211	< .001

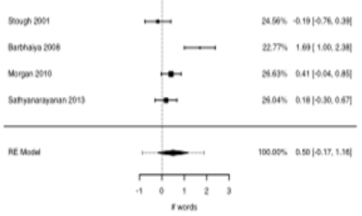

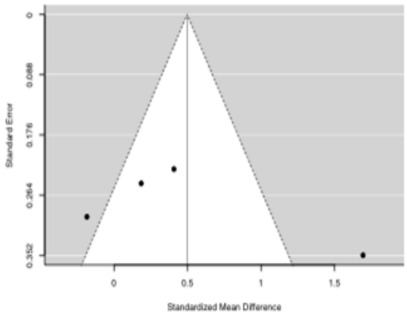

AVLT Forgetting rate

Random-Effects Model (k = 3)

	Estimate	se	Z	p	CI Lower Bound	CI Upper Bound
Intercept	-0.0272	0.145	-0.188	0.851	-0.311	0.256
	.	.	.	.	.	.

Note. Tau² Estimator: DerSimonian-Laird

Heterogeneity Statistics

Tau	Tau²	I²	H²	R²	df	Q	p
0.000	0 (SE= 0.0644)	0%	1.000	.	2.000	1.584	0.453

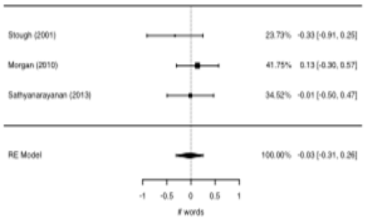

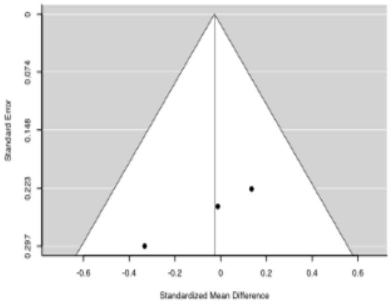

AVLT Proactive interference

Random-Effects Model (k = 3)

	Estimate	se	Z	p	CI Lower Bound	CI Upper Bound
Intercept	0.620	0.343	1.81	0.071	-0.052	1.292
	.	.	.	.	.	.

Note. Tau² Estimator: DerSimonian-Laird

Heterogeneity Statistics

Tau	Tau²	I²	H²	R²	df	Q	p
0.532	0.283 (SE= 0.3548)	80.41%	5.106	.	2.000	10.212	0.006

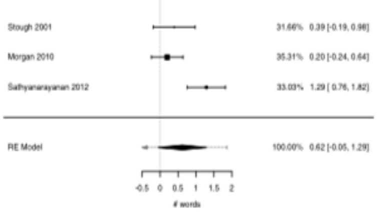

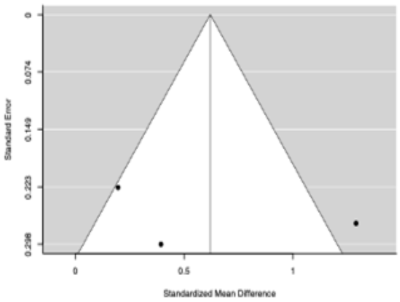

AVLT Retroactive Interference

Random-Effects Model (k = 3)

	Estimate	se	Z	p	CI Lower Bound	CI Upper Bound
Intercept	-0.129	0.225	-0.575	0.565	-0.571	0.312
	.	.	.	.	.	.

Note. Tau² Estimator: DerSimonian-Laird

Heterogeneity Statistics

Tau	Tau²	I²	H²	R²	df	Q	p
0.294	0.0867 (SE= 0.1524)	57.17%	2.335	.	2.000	4.669	0.097

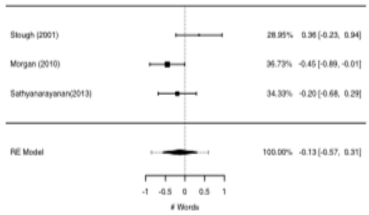

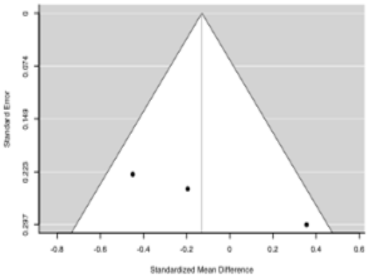

Digit span Forwards

Random-Effects Model (k = 5)

	Estimate	se	Z	p	CI Lower Bound	CI Upper Bound
Intercept	0.0710	0.444	0.160	0.873	-0.799	0.941
	.	.	.	.	.	.

Note. Tau² Estimator: DerSimonian-Laird

Heterogeneity Statistics

Tau	Tau²	I²	H²	R²	df	Q	p
0.940	0.8841 (SE= 0.7133)	90.28%	10.287	.	4.000	41.147	< .001

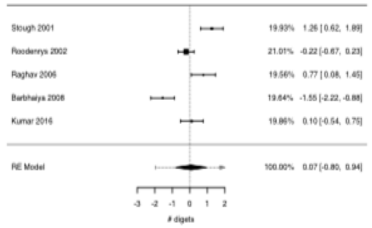

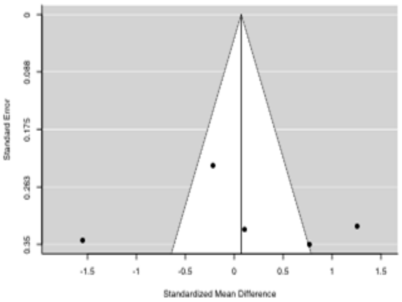

Digit Span Reverse

Random-Effects Model (k = 5)

	Estimate	se	Z	p	CI Lower Bound	CI Upper Bound
Intercept	-0.0375	0.285	-0.132	0.895	-0.595	0.520
	.	.	.	.	.	.

Note. Tau² Estimator: DerSimonian-Laird

Heterogeneity Statistics

Tau	Tau²	I²	H²	R²	df	Q	p
0.560	0.3142 (SE= 0.2894)	78.19%	4.585	.	4.000	18.341	0.001

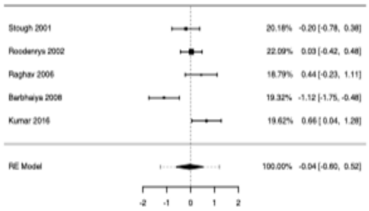

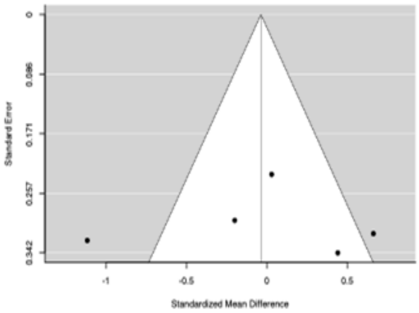

Paired associate learning

Random-Effects Model (k = 3)

	Estimate	se	Z	p	CI Lower Bound	CI Upper Bound
Intercept	0.308	0.210	1.47	0.143	-0.104	0.719
	.	.	.	.	.	.

Note. Tau² Estimator: DerSimonian-Laird

Heterogeneity Statistics

Tau	Tau²	I²	H²	R²	df	Q	p
0.163	0.0264 (SE= 0.1327)	19.9%	1.248	.	2.000	2.497	0.287

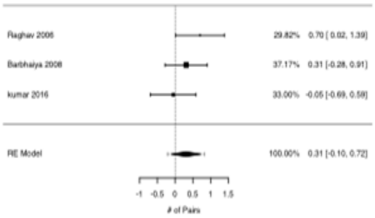

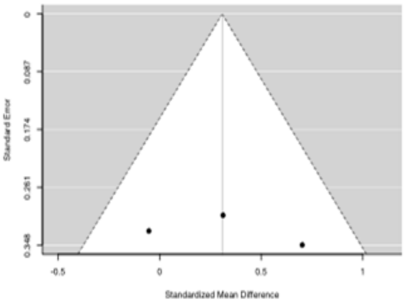

Simple Reaction time

Random-Effects Model (k = 3)

	Estimate	se	Z	p	CI Lower Bound	CI Upper Bound
Intercept	0.308	0.210	1.47	0.143	-0.104	0.719
	.	.	.	.	.	.

Note. Tau² Estimator: DerSimonian-Laird

Heterogeneity Statistics

Tau	Tau²	I²	H²	R²	df	Q	p
0.163	0.0264 (SE= 0.1327)	19.9%	1.248	.	2.000	2.497	0.287

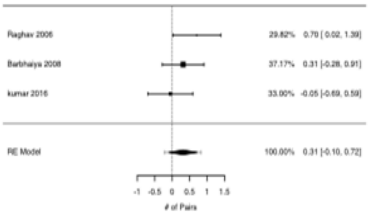

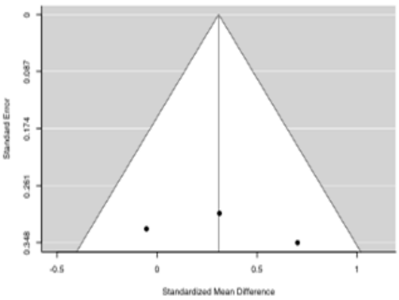

Choice Reaction Time

Random-Effects Model (k = 4)

	Estimate	se	Z	p	CI Lower Bound	CI Upper Bound
Intercept	-0.199	0.148	-1.34	0.179	-0.490	0.092
	.	.	.	.	.	.

Note. Tau² Estimator: DerSimonian-Laird

Heterogeneity Statistics

Tau	Tau²	I²	H²	R²	df	Q	p
0.000	0 (SE= 0.0728)	0%	1.000	.	3.000	2.269	0.518

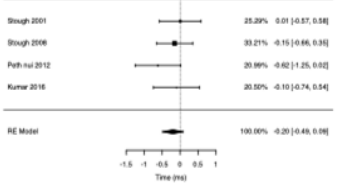

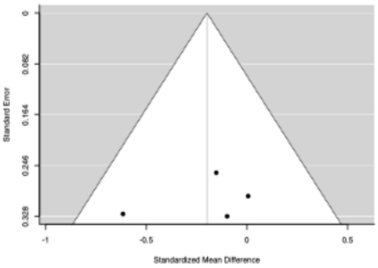

Logical Memory

Random-Effects Model (k = 3)

	Estimate	se	Z	p	CI Lower Bound	CI Upper Bound
Intercept	1.11	0.349	3.18	0.001	0.425	1.795
	.	.	.	.	.	.

Note. Tau² Estimator: DerSimonian-Laird

Heterogeneity Statistics

Tau	Tau²	I²	H²	R²	df	Q	p
0.517	0.2669 (SE= 0.3677)	73.23%	3.735	.	2.000	7.470	0.024

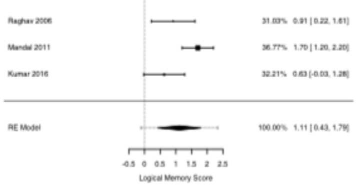

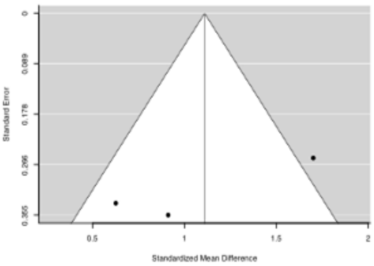

Supplement: Supplementary file 2 — Supplementary Information 2. [file 41598_2020_80045_MOESM2_ESM.pdf]
